# Familial Ovarian Cancer Clusters with Other Cancers

**DOI:** 10.1038/s41598-018-29888-4

**Published:** 2018-08-01

**Authors:** Guoqiao Zheng, Hongyao Yu, Anna Kanerva, Asta Försti, Kristina Sundquist, Kari Hemminki

**Affiliations:** 1 Division of Molecular Genetic Epidemiology, German Cancer Research Center (DKFZ), Heidelberg, Germany; 2 Faculty of Medicine, University of Heidelberg, Heidelberg, Germany; 3 Cancer Gene Therapy Group, Faculty of Medicine, University of Helsinki, Helsinki, Finland; 4 Department of Obstetrics and Gynecology, Helsinki University Hospital, Helsinki, Finland; 5 Center for Primary Health Care Research, Lund University, 205 02, Malmö, Sweden; 6 Department of Family Medicine and Community Health, Department of Population Health Science and Policy, Icahn School of Medicine at Mount Sinai, New York, USA; 7 Center for Community-based Healthcare Research and Education (CoHRE), Department of Functional Pathology, School of Medicine, Shimane University, Izumo, Japan

## Abstract

Familial risk of ovarian cancer is well-established but whether ovarian cancer clusters with other cancers and the clusters differ by histology remains uncertain. Using data from the Swedish Family-Cancer Database, we explored familial associations of ovarian cancer with other cancers with a novel approach; relative risk for (histology-specific) ovarian cancer was estimated in families with patients affected by other cancers, and conversely, risks for other cancers in families with (histology-specific) ovarian cancer patients. Eight discordant cancers were associated with ovarian cancer risk, of which family history of breast cancer showed a dose-response (P-trend <0.0001). Conversely, risks of eight types of cancer increased in families with ovarian cancer patients, and dose-responses were shown for risks of liver (P-trend = 0.0083) and breast cancers (P-trend <0.0001) and cancer of unknown primary (P-trend = 0.0157). Some cancers were only associated with histology-specific ovarian cancers, e.g. endometrial cancer was only associated with endometrioid type but with highest significance. Novel associations with virus-linked cancers of the nose and male and female genitals were found. The results suggest that ovarian cancer shares susceptibility with a number of other cancers. This might alert genetic counselors and challenge approaches for gene and gene-environment identification.

## Introduction

Ovarian cancer is a heterogeneous disease commonly classified into epithelial and non-epithelial types. Histologically, epithelial ovarian cancer includes high-grade serous, low-grade serous, endometrioid, clear cell and mucinous carcinoma^1^. Non-epithelial ovarian cancer, which accounts for less than 10% of all ovarian cancer cases^2^, comprises many tumor types such as granulosa cell and germinal malignancies, teratomas, and dysgerminomas; we refer to these as non-epithelial tumors. Distinctive susceptibility to protective and risk factors such as oral contraceptives, endometriosis, and smoking has been observed for different histological types of ovarian cancer^3^. Furthermore, histology-specific ovarian cancers present different prognoses. For example,patient with high-grade serous ovarian carcinoma always has poor prognosis compared to other types because most patients (∼80%) present with advanced stage at diagnosis^1^.

It is well-known that family history of ovarian cancer is associated with increased ovarian cancer risk and the relative risk is estimated to be 2.0 to 4.0 when having a first-degree relative affected by ovarian cancer^4–8^. The most common genes predisposing to ovarian cancer are *BRCA1/2*, which are associated with high-grade serous histology^9,10^. Mutations in *mismatch repair (MMR)* genes, which are responsible for the hereditary nonpolyposis colorectal cancer (HNPCC) syndrome, also contribute to the familial ovarian cancer with a tendency towards endometriod and clear cell histology^11,12^. Some other moderate and low penetrant genes, including *BRIP1*, *RAD51C* and *RAD51D* may also contribute to ovarian cancer risk^13^. All high- and moderate risk genes predisposing to ovarian cancer also increase risks for other, i.e. discordant, cancers. For instance, *BRCA1/2* mutations predispose to breast, prostate, pancreatic and some other cancers^14^; *MMR* gene mutations predispose to colorectal, endometrial and stomach cancers^15^; and rare *PALB2* mutations predispose to breast and prostate cancer^16,17^. At the population level, discordant associations of ovarian cancer have been observed with breast, endometrial and prostate cancers^18–20^, and also with some other cancers with lower statistical significance^21,22^. There are very few studies concerning associations of histological specific ovarian cancer with other cancers and our own work dates a decade back when the Swedish Family-Cancer Database was far from its present size^18^. Our present study covers 8,850 patients with histological information until the end of 2015, doubling the power of detection compared to the previous study, which included 4,082 such cases^18^.

The aim of our present study was to explore the familial associations of histology-specific ovarian cancer with other discordant cancers using multiple independent analyses for reliable results. The patient population includes 46,227 ovarian cancer patients and 10,639 other cancer patients who had a family history of ovarian cancer in first degree relatives. Our results should be relevant for genetic counseling, precise treatment and health care for patients with ovarian cancer and may provide clues about shared genetic and/or environmental risk factors.

## Results

A total of 46,227 ovarian cancer patients were found in the database, of which 11,301 were diagnosed in the offspring generation at the median age of 63-years-old (Table 1). There were 8,850 ovarian cancer patients in the offspring generation diagnosed since 1993 when Systemized Nomenclature of Medicine (SNOMED) codes were applied, and 11.9% of them were non-epithelial ovarian cancers. In the offspring generation, there were 4526 (40.0%) patients who had one first-degree relative affected by any discordant cancer and 2395 (21.2%) patients had at least two first-degree relatives affected by any discordant cancer. For ovarian cancer alone, there were 467 (4.3%) patients who had one first-degree relative affected by ovarian cancer and 20 (0.2%) patients that had two affected first-degree relatives.

**Table 1:** Characteristics of ovarian cancer patients in offspring generation (1958–2015).

No. of females followed		4,216,676
No. of ovarian cancer patients		11,301
No. of ovarian cancer patients (1993–2015)		8850
Median age at diagnosis of ovarian cancer		63 years old
No. of ovarian cancer patients with family history of ovarian cancer	In one first-degree relative	467 (4.3%)
	In at least two first-degree relatives	20 (0.2%)
No. of ovarian cancer patients with family history of discordant cancers	In one first-degree relative	4526 (40.0%)
	In at least two first-degree relatives	2395 (21.2%)
Histological types (1993–2015)	Undifferentiated	193 (2.2%)
	Clear cell	511 (5.8%)
	Endometrioid	999 (11.3%)
	Serous	4149 (46.9%)
	Mucinous	726 (8.2%)
	Non-epithelial	1053 (11.9%), 300 of them were thecoma
	Others	1219 (13.8%)

Others include histological types of other ovarian cancers such as papillary ovarian cancer, as well as unspecified ovarian cancers.

**Figure 1:**
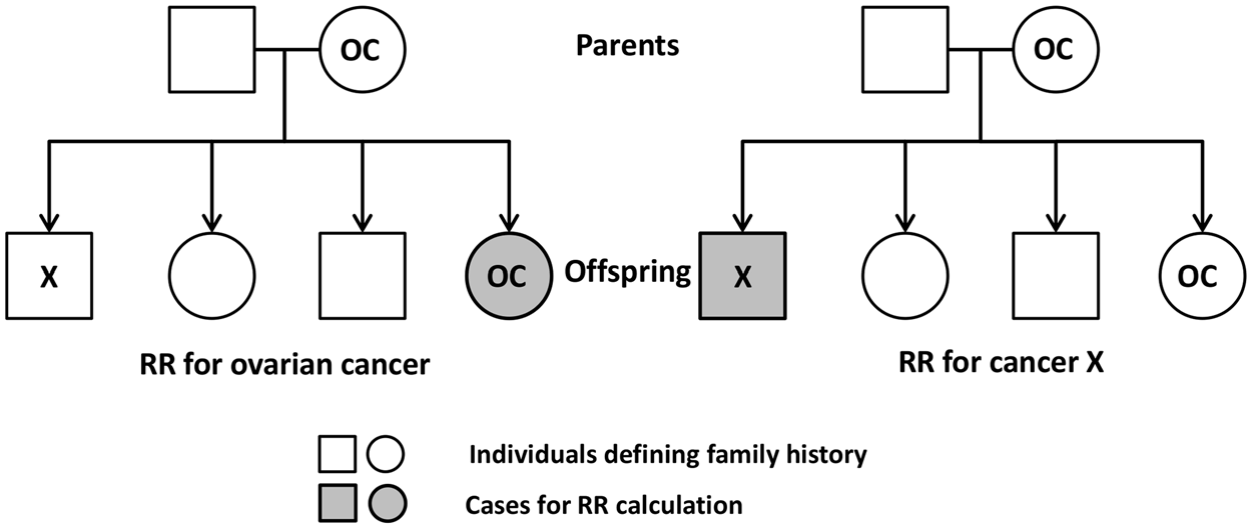
Flowchart of calculating the RRs for ovarian cancer and cancer X in a two-way analysis. On the left side, RR was calculated for ovarian cancer when family history was cancer X; person-years at risk were calculated for all offspring; probands were all first-degree relatives. On the right side, RR was calculated for cancer X when family history was ovarian cancer. OC: ovarian cancer.

The principles of the bi-directional analyses are shown in Fig. 1. On the left side, RR is calculated for ovarian cancer (OC); person-years at risk are calculated for all persons in the offspring generation; probands are all first-degree relatives with cancer X. In the reverse analysis, (on the right side) RR is calculated for cancer X when first-degree relatives have ovarian cancer. For parent-offspring generations, the pairs of individuals are independent between the two analyses but for siblings the pairs of individuals are the same and thus not completely independent.

**Figure 2:**
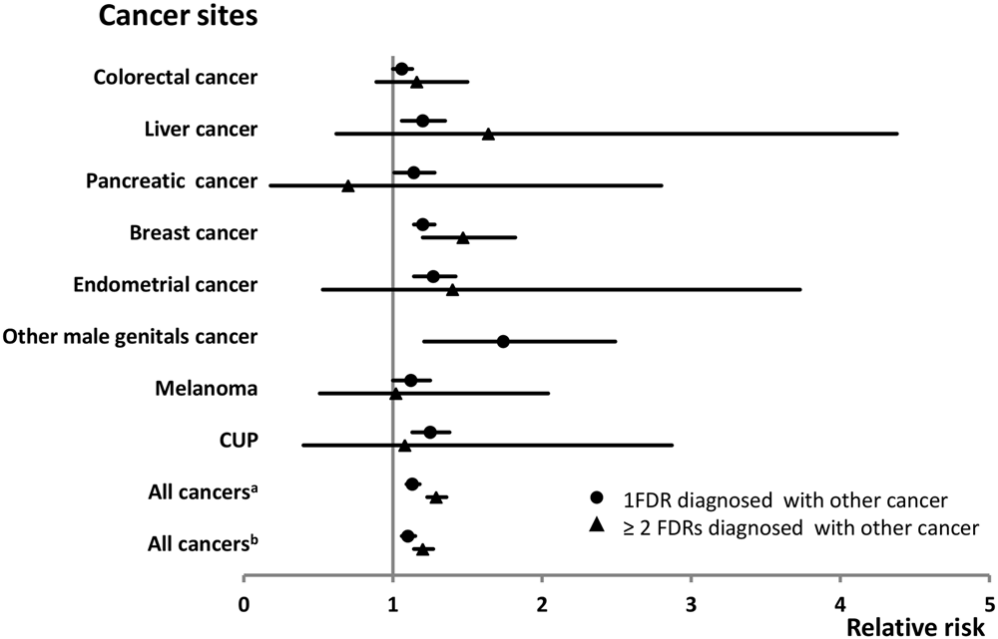
Ovarian cancer risk when only one or at least two first-degree relatives were diagnosed with other cancers. Only significant cancer sites are shown. CUP: cancer of unknown primary; ^a^all cancers include ovarian cancers and all other cancers; ^b^all cancers include all other cancers except ovarian cancer. FDR, first-degree relative.

Table 2 shows the invasive ovarian cancer risk when first-degree relatives were diagnosed with ovarian cancer or other cancers and cancer sites with significant results are shown in the forest plot (Fig. 2). The cancer sites with less than 30 cases having affected first-degree relative and insignificant results are not displayed in Table 2 nor in the following Table. The relative risk of ovarian cancer was 2.42 in families with one ovarian cancer patient and it reached 11.36 with two affected first-degree relatives, both of which were significant at a 0.001 level. For discordant cancer, eight cancers showed  significant results in the associations with the risk of ovarian cancer. Family history of breast cancer showed a dose-response on ovarian cancer risk (P-trend test <0.001). RR was 1.20 when one first-degree relative was diagnosed with breast cancer (P < 0.001) and RR was 1.47 when two first-degree relatives were affected (P < 0.01). Ovarian cancer risk increased in families with one first-degree relative diagnosed with colorectal (1.06), liver (1.20, P < 0.01), pancreatic (1.14) and endometrial (RR 1.27, P < 0.001) cancers, melanoma (1.12) and cancer of unknown primary (CUP, 1.25 P < 0.001). For liver cancer subtypes (N = 277), we observed increased risk of ovarian cancer in families of patients with gallbladder cancer (N = 95, 34.3% of all liver cancer; RR = 1.27, 95%CI 1.03–1.55; see Supplementary Table S1). Risk of ovarian cancer was found to be elevated in families that had one patient diagnosed with any cancer (1.13), and RR was 1.29 when the families had at least two cancer patients. When only considering the family history of discordant cancers, we found the risk of ovarian cancer was still significantly increased. Statistical power to detect a significant association is shown in Table 2. This is calculated for two-sided confidence level of 95% and an RR of 1.20 for one first-degree relative affected by other cancer and an RR of 1.40 when at least two first-degree relatives were affected. In families of one affected first-degree relative, an 80% power was reached only for  colorectal, lung, breast and prostate cancers. In families of more than one first-degree relative affected, no association reached a power of 80% at an RR of 1.4.

**Table 2:** Invasive ovarian cancer risk when first-degree relatives were diagnosed with other cancers.

Cancer site	Patients with 1 FDR				Patients with ≥ 2 FDRs				*P-trend*
	*N*	*RR*	*95%CI*	*Power (%) RR* = *1*.*2*	*N*	*RR*	*95%CI*	*Power (%) RR* = *1*.*4*	
Ovary	467	***2***.***42***	2.21–2.66	46.6	20	***11***.***36***	7.33–17.62	8.2	<0.0001
Upper aerodigestive tract	239	1.08	0.95–1.23	52.7	4	1.46	0.55–3.90	9.1	0.1887
Esophagus	70	0.94	0.74–1.19	22.8	0	—	—	—	0.6042
Stomach	349	1.08	0.97–1.2	56.4	3	0.57	0.18–1.76	10.5	0.2887
Small intestine	41	1.02	0.75–1.38	16.3	1	4.07	0.57–28.93	37.7	0.7269
Colorectum	1009	**1**.**06**	1.00–1.13	96.7	58	1.16	0.89–1.50	37.7	0.0377
Colon	659	1.04	0.96–1.13	88.3	28	1.33	0.92–1.92	22.0	0.141
Rectum	395	1.07	0.97–1.19	69.7	10	1.48	0.80–2.76	12.3	0.0974
Liver	277	***1***.***20***	1.06–1.35	48.9	4	1.64	0.62–4.38	8.6	0.0024
Pancreas	271	**1**.**14**	1.01–1.28	50.4	2	0.70	0.18–2.80	9.1	0.0595
Lung	655	1.05	0.97–1.14	89.6	27	1.04	0.71–1.52	26.3	0.2382
Breast	1243	***1***.***20***	1.14–1.28	99.2	88	***1***.***47***	1.20–1.82	43.8	<0.0001
Cervix	184	1.14	0.98–1.32	45.1	0	—	—	—	0.0862
Endometrium	317	***1***.***27***	1.14–1.42	54.4	4	1.40	0.53–3.73	9.0	<0.0001
Other female genitals	39	0.94	0.69–1.29	15.0	0	—	—	—	0.7144
Prostate	1320	1.02	0.96–1.08	99.6	121	1.14	0.95–1.36	56.2	0.2174
Testis	35	1.20	0.86–1.68	23.8	1	4.37	0.62–31.03	6.7	0.1904
Other male genitals	30	***1***.***74***	1.21–2.49	10.4	0	—	—	—	0.0056
Kidney	276	1.08	0.96–1.22	56.2	3	0.89	0.29–2.75	9.6	0.2458
Bladder	403	0.96	0.87–1.06	75.5	14	1.57	0.93–2.66	13.7	0.8195
Melanoma	339	**1**.**12**	1.00–1.25	78.4	8	1.02	0.51–2.04	16.1	0.0573
Skin	387	0.98	0.88–1.08	72.4	12	1.20	0.68–2.11	14.8	0.8309
Nervous system	279	1.08	0.96–1.22	68.4	2	0.49	0.12–1.97	11.3	0.2972
Thyroid gland	75	1.04	0.83–1.31	29.0	0	—	—	—	0.7147
Endocrine glands	148	0.97	0.83–1.14	45.5	1	0.72	0.10–5.10	8.2	0.6948
Connective tissue	61	1.06	0.83–1.37	21.9	0	—	—	—	0.6436
Non-Hodgkin lymphoma	296	1.08	0.96–1.21	63.6	5	1.27	0.53–3.05	10.5	0.1882
Hodgkin lymphoma	52	1.28	0.97–1.68	20.0	0	—	—	—	0.0887
Myeloma	141	1.07	0.90–1.26	33.6	0	—	—	—	0.4464
Leukemia	250	0.99	0.87–1.12	60.7	2	0.50	0.13–2.01	10.4	0.6704
CUP	396	***1***.***25***	1.13–1.38	62.4	4	1.08	0.40–2.87	10.0	<0.0001
All cancers^a^	4553	***1***.***13***	1.09–1.18	100.0	2589	***1***.***29***	1.23–1.36	100.0	<0.0001
All cancers^b^	4340	***1***.***10***	1.06–1.15	100.0	2315	***1***.***20***	1.14–1.27	100.0	<0.0001

FDR: first-degree relative; CUP: cancer of unknown primary.

Bolding, italic and underlining indicate that the 95% CI, 99% CI and 99.9% CI did not overlap with 1.00 respectively.

^a^all cancers include ovarian cancers and all other cancers.

^b^all cancers include all other cancers except ovarian cancer.

**Figure 3:**
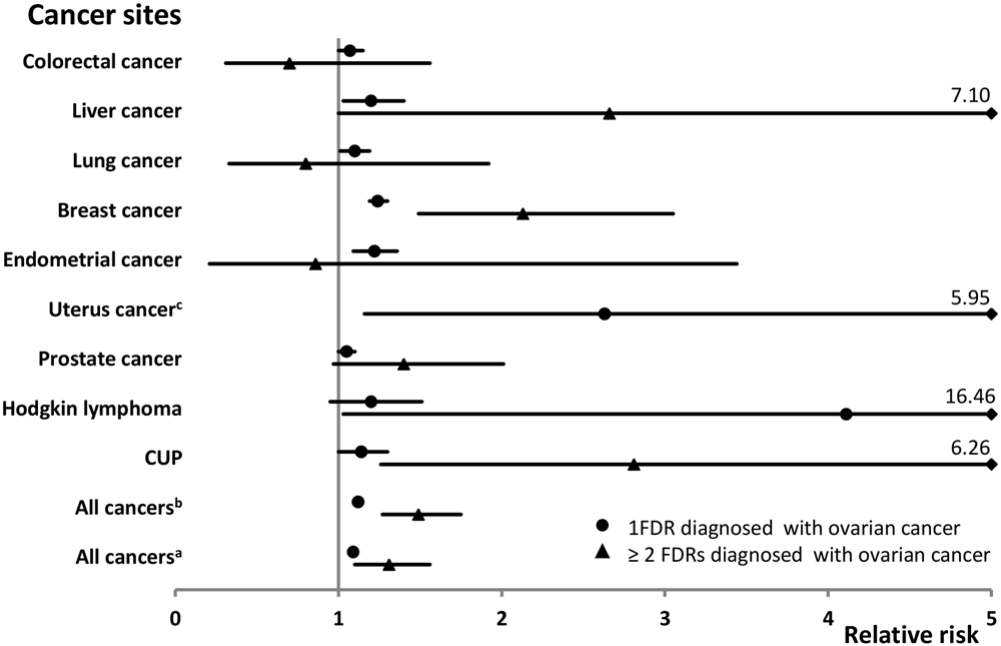
Other cancer risk when only one or at least two first-degree relatives were diagnosed with ovarian cancer. Only significant cancer sites are shown. CUP: cancer of unknown primary; ^a^all cancers include ovarian cancers and all other cancers; ^b^all cancers include all other cancers except ovarian cancer. FDR, first-degree relative; ^c^cancer of other parts of uterus, including chorionepithelioma.

In the reverse comparison, the overall cancer risk in the offspring generation increased when having a family history of ovarian cancer (Table 3). All the significant cancer sites are displayed in Fig. 3. The estimated RR was 1.12 (P < 0.001) in families of one first-degree relative affected by ovarian cancer and it was 1.49 (P < 0.001) when at least two were affected. Excluding ovarian cancer, the RRs for any other cancer were 1.09 (P < 0.001) and 1.31(P < 0.01), respectively. Five cancers (colorectal, liver, breast, endometrial cancers and CUP) were observed with significant results in the two-way comparison and family history of ovarian cancer showed dose-response effect on the risk of liver and breast cancers and CUP. Notably, when at least two first-degree relatives were diagnosed with ovarian cancer, the relative risk for Hodgkin lymphoma reached 4.11 (P < 0.01). However, that association was based on two families. Elevated risks were also observed for lung (1.10) and prostate (1.06) cancer when one first-degree relative was affected by ovarian cancer. Risk of esophageal (0.76) cancer declined in families with one first-degree relative diagnosed with ovarian cancer. In families of one first-degree relative diagnosed with ovarian cancer, an 80% power (RR of 1.2) was reached by colorectal, lung, breast and prostate cancers and by melanoma. In families with more than one first-degree relative affected, no association reached a power of 80% (1.4).

**Table 3:** Other cancer risk when first-degree relatives were diagnosed with invasive ovarian cancer.

Cancer site	Patients with 1 FDR				Patients with ≥2 FDRs				*P-trend*
	*N*	*RR*	*95%CI*	*Power (%) RR* = *1*.*2*	*N*	*RR*	*95%CI*	*Power (%) RR* = *1*.*4*	
Upper aerodigestive tract	220	1.02	0.89–1.17	49.3%	3	1.32	0.43–4.09	8.5	0.7011
Esophagus	52	**0**.**76**	0.58–1.00	20.5	1	1.29	0.18–9.16	6.6	0.0552
Stomach	134	1.11	0.94–1.32	31.3	3	2.29	0.74–7.10	7.4	0.1435
Small intestine	54	1.29	0.98–1.68	15.4	0	—	—	—	0.0793
Colorectum	884	**1**.**07**	1.00–1.15	95.1	6	0.70	0.31–1.56	13.4	0.0708
Colon	552	1.06	0.98–1.16	83.0	2	0.38	0.09–1.50	11.1	0.2651
Rectum	332	1.09	0.97–1.21	61.1	4	1.23	0.46–3.27	9.2	0.1264
Liver	167	**1**.**20**	1.03–1.40	35.2	4	**2**.**66**	1.00–7.10	7.6	0.0083
Pancreas	157	0.97	0.82–1.13	38.9	2	1.14	0.28–4.54	7.8	0.6918
Lung	634	**1**.**10**	1.01–1.19	85.4	5	0.80	0.33–1.92	11.4	0.0320
Breast	1981	***1***.***24***	1.19–1.30	99.9	30	***2***.***13***	1.49–3.05	19.5	<0.0001
Cervix	165	1.08	0.92–1.26	43.3	2	1.65	0.41–6.61	7.9	0.3036
Endometrium	304	***1***.***22***	1.09–1.36	53.8	2	0.86	0.21–3.44	8.5	0.0014
Uterus^c^	6	**2**.**63**	1.16–5.95	5.6	0	—	—	—	0.0439
Other female genitals	27	0.89	0.61–1.30	13.0	0	—	—	—	0.5488
Prostate	1727	**1**.**05**	1.00–1.10	99.9	29	1.40	0.97–2.01	20.2	0.0149
Testis	117	1.15	0.95–1.37	38.0	0	—	—	—	0.1551
Other male genitals	22	1.16	0.76–1.77	10.1	0	—	—	—	0.5002
Kidney	226	1.10	0.96–1.25	48.4	5	2.30	0.96–5.53	8.4	0.0905
Bladder	333	0.99	0.89–1.10	64.7	4	1.07	0.40–2.85	9.5	0.8401
Melanoma	628	1.07	0.99–1.16	89.7	7	1.29	0.62–2.71	12.1	0.0645
Skin	333	1.06	0.95–1.18	62.5	2	0.62	0.16–2.49	9.3	0.3549
Nervous system	380	1.04	0.94–1.15	77.9	3	0.89	0.29–2.77	10.6	0.4900
Thyroid gland	111	1.11	0.92–1.34	31.7	1	1.18	0.17–8.35	7.4	0.2767
Endocrine glands	193	1.01	0.88–1.16	48.0	2	1.13	0.28–4.51	8.4	0.8776
Connective tissue	70	1.01	0.79–1.28	23.0	0	—	—	—	0.4857
Non-Hodgkin lymphoma	327	1.11	0.99–1.24	63.2	3	0.99	0.32–3.08	9.4	0.0751
Hodgkin lymphoma	71	1.20	0.95–1.51	25.0	2	**4**.**11**	1.03–16.46	7.0	0.0732
Myeloma	107	1.09	0.90–1.32	26.5	0	—	—	—	0.3765
Leukemia	248	0.98	0.86–1.11	63.4	3	1.22	0.39–3.79	9.4	0.8095
CUP	227	**1**.**14**	1.00–1.30	45.8	6	**2**.**81**	1.26–6.26	8.3	0.0157
All cancers^a^	10492	***1***.***12***	1.10–1.14	100.0	147	***1***.***49***	1.27–1.75	68.7	<0.0001
All cancers^b^	10025	***1***.***09***	1.07–1.11	100.0	127	***1***.***31***	1.10–1.56	67.9	<0.0001

FDR: first-degree relative; CUP: cancer of unknown primary.

Bolding, italic and underlining indicate that the 95% CI, 99% CI and 99.9% CI did not overlap with 1.00 respectively.

^a^all cancers include ovarian cancers and all other cancers.

^b^all cancers include all other cancers except ovarian cancer.

^c^cancer of other parts of uterus, including chorionepithelioma.

In the search of the familial associations of histology-specific ovarian cancer with other cancers, some significant results were observed (Table 4; note that both of the two-way comparisons are shown). The significant pairs (histology type-cancer site) in the two-way analysis were clear cell-pancreas (joint P < 0.0025), endometrioid-nose (joint P < 0.0025), endometrioid-breast (joint P < 0.0005), endometrioid- endometrium (joint P < 10^−6^), serous-breast (joint P < 10^−6^), serous-male genitals (joint P < 5 × 10^−5^), mucinous-gallbladder (joint P < 10^−4^; see Supplementary Table S2). For the above pair ‘endometrioid-endometrium’, 40 of 999 patients with endometrioid ovarian cancer were diagnosed within five months with endometrial cancer thus resulting in an RR of 9.49 (6.96–12.95) for endometrial cancer compared to the risk of first endometrial cancer. Conversely, 164 patients were diagnosed with ovarian cancer among the 12,294 patients with endometrial cancer, and 76 of these were of endometrioid histology; more than half of these were diagnosed in the same month as those with endometrial cancer. The RR of endometrioid ovarian cancer after endometrial cancer was 20.30 (15.92–25.90, see Supplementary Table S3). For the pair endometrioid-nose, the available histology for cancer of nose included two squamous cell carcinomas and one adenocarcinomas. Of note, results for non-epithelial ovarian cancer were all based on a small number of cases.

**Table 4:** Familial associations of histology-specific ovarian cancer with other cancers.

Histology	Cancer site	Risk of ovarian cancer				Risk of other cancer			
		*N*	*RR*	*95% CI*	*Power (%) RR* = *1*.*4*	*N*	*RR*	*95% CI*	*Power (%) RR* = *1*.*4*
Undifferentiated	Stomach	11	**1**.**95**	1.06–3.60	53.4	2	1.65	0.41–6.61	8.3
	Liver	8	2.00	0.99–4.06	46.7	4	**2**.**76**	1.04–7.36	8.8
	Pancreas	5	1.16	0.48–2.81	48.2	7	***4***.***07***	1.94–8.55	9.2
	Lung	17	1.48	0.90–2.44	86.3	11	**1**.**85**	1.02–3.34	14.8
	Hodgkin lymphoma	3	**4**.**35**	1.39–13.61	20.9	1	2.35	0.33–16.71	7.2
Clear cell	Pancreas	18	**1**.**69**	1.05–2.70	51.9	7	**2**.**23**	1.06–4.68	11.1
	Testis	3	2.19	0.70–6.81	26.3	6	***3***.***94***	1.77–8.76	9.7
Endometrioid	Stomach	35	1.25	0.89–1.75	63.2	11	**2**.**15**	1.19–3.88	13.8
	Nose	4	**3**.**08**	1.15–8.21	12.1	2	**4**.**02**	1.00–16.10	7.0
	Lung	58	0.98	0.75–1.28	93.3	37	**1**.**43**	1.04–1.97	35.1
	Breast	121	**1**.**24**	1.02–1.49	99.6	94	***1***.***35***	1.10–1.65	67.3
	Endometrium	49	***2***.***22***	1.67–2.96	61.0	22	***2***.***04***	1.34–3.09	20.3
	Other female genitals	6	1.65	0.74–3.69	18.7	4	**3**.**08**	1.16–8.22	8.5
	Kidney	34	**1**.**50**	1.07–2.12	63.1	12	1.41	0.80–2.49	18.2
	Connective tissue	8	1.54	0.77–3.09	26.7	6	**2**.**53**	1.13–5.63	10.8
Serous	Breast	515	***1***.***27***	1.15–1.39	100.0	373	***1***.***24***	1.12–1.38	58.9
	Other male genitals	12	**1**.**87**	1.06–3.29	18.3	8	***2***.***42***	1.20–4.85	11.6
	Thyroid gland	38	**1**.**42**	1.03–1.96	53.9	14	0.89	0.53–1.51	27.5
	CUP	134	**1**.**23**	1.04–1.47	87.6	39	1.06	0.77–1.45	47.2
Mucinous	Upper aerodigestive tract	23	***1***.***73***	1.14–2.63	56.7	11	1.70	0.94–3.08	16.5
	Nose	3	**3**.**43**	1.10–10.67	11.7	0	—	—	—
	Breast	83	1.23	0.98–1.54	99.4	67	***1***.***38***	1.08–1.75	58.9
	Bladder	37	**1**.**46**	1.05–2.04	60.1	11	1.15	0.64–2.08	16.2
Non-epithelial	Thyroid gland	5	**2**.**82**	1.17–6.48	29.9	1	1.20	0.17–8.50	8.3
	Connective tissue	5	***3***.***69***	1.52–8.94	23.2	1	1.81	0.25–12.84	7.7
	Non-Hodgkin lymphoma	6	0.90	0.40–2.02	61.7	7	***2***.***72***	1.30–5.71	12.3
	CUP	14	***2***.***25***	1.31–3.86	56.0	3	1.66	0.54–5.15	10.6

CUP: cancer of unknown primary.

Bolding, italic and underlining indicate that the 95% CI, 99% CI and 99.9% CI did not overlap with 1.00 respectively.

^a^all cancers include ovarian cancers and all other cancers.

^b^all cancers include all other cancers except ovarian cancer.

Table 5 displays familial association of histology-specific ovarian cancer with any cancer. Undifferentiated, endometrioid, serous and mucinous ovarian cancers were significantly associated with any cancers, among which undifferentiated (joint P < 0.0025), endometrioid (joint P < 10^−6^) and serous (joint P < 5 × 10^−5^) types showed significant associations in the two-way comparison. After omitting ovarian cancer, results for endometrioid, serous and mucinous types were still significant in the associations with any discordant cancers and the association of endometrioid carcinoma was significant in the two-way comparison (joint P < 10^−5^).

**Table 5:** Familial associations of histology-specific ovarian cancer with any cancer.

Histology	Cancer site	Risk of ovarian cancer				Risk of other cancer			
		*N*	*RR*	*95% CI*	*Power (%) RR* = *1*.*4*	*N*	*RR*	*95% CI*	*Power (%) RR* = *1*.*4*
Undifferentiated	All cancers^a^	134	**1**.**45**	1.07–1.97	37.3	119	**1**.**23**	1.03–1.48	82.6
	All cancers^b^	116	1.29	0.94–1.77	36.9	110	1.16	0.96–1.40	81.9
Clear cell	All cancers^a^	302	0.99	0.82–1.18	84.8	168	1.01	0.87–1.18	96.2
	All cancers^b^	287	0.96	0.80–1.15	84.2	161	0.99	0.85–1.16	95.9
Endometrioid	All cancers^a^	675	***1***.***41***	1.23–1.61	95.7	477	***1***.***21***	1.11–1.32	100.0
	All cancers^b^	627	***1***.***34***	1.17–1.54	95.4	448	***1***.***16***	1.06–1.27	100.0
Serous	All cancers^a^	2663	***1***.***19***	1.12–1.27	100.0	1781	**1**.**04**	1.00–1.09	100.0
	All cancers^b^	2448	***1***.***13***	1.04–1.23	100.0	1711	1.02	0.98–1.07	100.0
Mucinous	All cancers^a^	439	**1**.**19**	1.02–1.38	93.6	290	1.05	0.94–1.18	99.9
	All cancers^b^	421	**1**.**17**	1.00–1.37	93.2	278	1.03	0.91–1.16	99.9
Non-epithelial	All cancers^a^	159	1.00	0.79–1.27	69.6	80	1.03	0.83–1.28	88.8
	All cancers^b^	154	1.00	0.79–1.27	68.9	76	1.00	0.80–1.25	88.2

Bolding, italic and underlining indicate that the 95% CI, 99% CI and 99.9% CI did not overlap with 1.00 respectively.

^a^all cancers include ovarian cancers and all other cancers.

^b^all cancers include all other cancers except ovarian cancer.

## Discussion

With novel insight from our bi-directional statistical analyses, we found that ovarian cancer was associated with a group of discordant cancers, among which colorectal, breast, endometrial and liver cancers and CUP were significant in the two-way analysis. As the present study involves multiple comparisons we require, for credible familial associations, that more than a single analysis should be positive and RRs should show a ‘dose-response’ relationship, i.e. increase by the number of affected probands. For guidance, we use the joint P-values. Breast cancer showed four significant RRs, of which three were at a 0.1% confidence level; liver cancer and CUP showed three increased RRs, and colorectal and endometrial cancers showed two RRs. For endometrial cancer, the two RRs were both significant at 0.001 levels. Cancer in other male genitals showed a single significant RR at a 1% confidence level. The remaining cancers, including lung and prostate cancers, melanoma and Hodgkin lymphoma showed a single nominal significance. The association of ovarian cancer with breast cancer showed dose-response in the two-way comparison, and the ones with liver cancer and CUP showed dose-response in the one-way analysis. The ‘dose-response’ observation in RRs would support the findings and it is informative of the underlying genetic risk (penetrance) as many first-degree relatives diagnosed with the same cancers signal high penetrance.

Ovarian cancer showed the strongest association with breast cancer (joint P < 10^−11^) and it may be attributable to *BRCA1/2* mutations as in the histological analysis breast cancer was associated with serous ovarian carcinomas, which have been reported to be related to *BRCA1/2* mutations^9,10^. *BRCA2* carriers have an increased risk of prostate cancer^14,23^, but one weak association was shown here. *BAP1* mutations manifest eye and cutaneous melanomas, ovarian cancers and several other cancers, and these may contribute to the associations with eye cancer (one of two was melanoma) and the weakly increased risk with cutaneous melanoma in Table 1^23,24^. In our study, the association of ovarian cancer with colorectal cancer was weak and no significant associations were observed with endometrioid or clear cell ovarian cancers, which may not support the HNPCC link. By contrast, we found that endometrioid ovarian cancer was strongly associated with endometrial cancer in the two-way comparison, which could imply association with HNPCC syndrome related to *MSH6* mutations in patients with the endometrioid and clear cell ovarian carcinomas^25^. Carriers with *MSH6* germline mutations appear to have a high risk of endometrial cancer but low risk of colorectal cancer^26^.

Ovarian cancer is a heterogeneous disease and different histological types of ovarian cancer have distinctive risk factors, genetic characteristics and prognosis. Accordingly, our results of histology-based analysis suggest that some specific histological types are associated only with specific cancers. Endometrial cancer was only associated with endometrioid ovarian cancer and this pair showed the most significance (RR > 2.00, joint P < 10^−6^) among all the histology-based associations. Most endometrioid ovarian cancers were synchronous with endometrial cancer, which suggests these two cancers share common risk factors. Furthermore, breast cancer was associated with many histological types of epithelial ovarian cancers (endometrioid, serous and mucinous) with homogenous RRs between 1.20–1.40; this may suggest that different epithelial ovarian cancers share the similar risks with breast cancer. Smoking is a risk factor of mucinous ovarian cancer and, accordingly, the associated cancers were mostly smoking-related, including cancers in upper aerodigestive tract, nose, breast, bladder and gallbladder^3^. The most consistent association of lung cancer was found with undifferentiated ovarian cancer.

Ovarian cancer was associated with liver cancer in the two-way comparison (joint P < 2.5 × 10^−5^). As liver cancer in the 7th revision of International Classification of Diseases (ICD-7) encompasses cancers in primary liver, gallbladder, extrahepatic bile ducts and ampulla of vater, we performed subtype analyses for liver cancer. The only significant association was with gallbladder cancer, and notably, with mucinous ovarian cancers in the two-way comparison. Some common risk factors, such as smoking and obesity, may contribute to the association between ovarian cancer and gallbladder cancer^3,27,28^.

We found some curious associations with rare cancers most notably with cancer of the nose, which showed a two-way association with endometrioid ovarian cancer and a one-way association with mucinous ovarian cancer. Some known risk factors for  cancer of the nose, such as smoking and wood dust exposure, are an unlikely explanation for this finding^29^. However, Epstein-Barr virus infection, particularly in transiently immunocompromised individuals, has been associated with cancer of the nose and this may be a plausible explanation for the present findings^30,31^. The other rare cancers associating with ovarian cancer were male and female genital cancers; the common denominator for which is squamous cell histology and human papilloma virus infection etiology^30,32,33^. An increased risk of Hodgkin lymphoma, another Epstein-Barr virus related cancer, was observed in two families for each with two or more patients with ovarian cancer^30^. A note of caution is warranted as the number of cases with family history was small and this number of virally related cancers can be associated with ovarian cancer by chance. Nevertheless, this is an interesting finding and can be a guidance for further study. Some possible mechanisms could be socioeconomic factors and pro-inflammatory effects of obesity^34^.

CUP is a fatal cancer which is diagnosed at a metastatic stage and no primary site can be found. Familial association of CUP with many primary cancers, including ovarian cancer, was reported previously^35^. In that report we hypothesized that the primary cancers in family members of CUP patients would indicate the location of the primary cancer from which CUP was originating. In the present study, ovarian cancer was associated with CUP in the two-way comparison (joint P < 2.5 × 10^−6^); the previous hypothesis predicted that the primary site for CUP may be the ovary.

Non-epithelial malignancies of the ovary account for around 10% of all ovarian cancers in our database and the risk factors and genetic characteristics of non-epithelial ovarian cancer are poorly understood^2^. In our study, non-epithelial ovarian cancers were associated with thyroid gland and connective tissue cancers, non-Hodgkin lymphoma and CUP, but the associations were based on a single significant RR each.

The main limitation of this study is that the patients with identifiable histology were diagnosed only after 1993 since the introduction of ICD-O/2 in the cancer registry. This affects familial risk estimates because 22 years of follow-up is short for intergenerational studies considering risks of both the parental and offspring generations. Furthermore, histological classification has not been updated to meet the current guidelines. For example, serous histology is now considered to be either low-grade or high-grade with different prognoses and molecular events/etiologies. Although, to the best of our knowledge, this study on familial associations between ovarian cancer with other cancers had the largest sample size ever reported, the data lack sufficient power in many comparisons. As indicated in the tables, only a few associations had 80% or higher power to detect significant results. This also limits use of techniques such as the Bonferroni correction in adjustments for multiple testing, as has been discussed^36^. Insufficient clinico-behavioral information, such as smoking, is also a caveat in the analysis since they can be construed as potential confounders. However, as we adjusted the data for socioeconomic factors, this reduces greatly the possible confounding by smoking^37^. We have no genetic information for the ovarian cancer patients and the explanation for associations between cancers are based on speculation, which should be considered with caution.

In summary, discordant cancers were by far more common (61.2%) in ovarian cancer families than multiple ovarian cancers (4.5%). We found that ovarian cancer was associated with a group of discordant cancers, among which colorectal, breast, endometrial and liver cancers and CUP were significant in the two-way analysis. Some cancers were only associated with specific histological types of ovarian cancers; for example endometrial cancer was only significant with endometrioid types, and breast cancer was associated with endometrioid, serous and mucinous with homogenous familial risks. The novel associations with cancer of nose and that of male and female genitals were noted but the common etiological mechanism remains to be established. Our results should have implications for genetic counseling, and they may provide clues about shared genetic and/or environmental risk factors of ovarian cancer with other cancers.

## Methods

The Swedish Family-Cancer Database is the combination of the Multigeneration Register, national Cancer Registry (started in 1958), national censuses and Cause of Death Register. It includes all Swedish residents born after 1931 (offspring generation) and their biological parents (parental generation). The latest version of the Swedish Family-Cancer Database contains 16.1 million individuals among which almost 2.0 million were cancer patients recorded to the end of 2015.

Most common primary cancers (35) and CUP were identified with the 3-digital codes of ICD-7. According to SNOMED codes which were available in the database since 1993, “80203” was classified as undifferentiated ovarian cancer, “83103” as clear cell, “83803” as endometrioid, “84413” and “84603” as serous, “84703” as mucinous, and “86203” as thecoma. Thecoma was used to represent non-epithelial ovarian cancer. The follow-up for cancer in offspring generation commenced from the beginning of 1958 (for histological analysis it was the beginning of 1993), the birth year, or the immigration year, whichever came latest. The follow-up was terminated when a person was diagnosed with cancer, emigrated or died, or at the end of 2015, whichever came first.

All patients had a complete family history (including both parents) and cancer data with full diagnostic detailed collected by the Cancer Registry. The number of first-degree relatives (parents and/or siblings) who were affected with cancer was considered as the family history. Relative risks (RRs), calculated for the offspring generation, were used as a measure of assessing familial risks by comparing incidence rates for persons with affected relatives to incidence rates for those whose relatives had no cancer. In the two-way comparison (Fig. 1), firstly, RR for ovarian cancer (or specific histological ovarian cancer) was calculated when family history was discordant cancer X, and then in the reverse order RR for cancer X was calculated when family history was ovarian cancer (or specific histological ovarian cancer). For parents and offspring (large majority of familial cases) these comparisons are independent but for siblings the pairs of cases are the same. Significant results in two-way analyses provide support for a true association but a lacking two-way association is not strong evidence against an association because age distributions and case numbers may differ between two-way analyses.

Poisson regression model was employed to estimate RRs and corresponding confidence intervals (CI) at 5%, 1% and 0.1% significance levels. These can be combined to calculate a joint significance for non-dependent associations; for example if the significant levels of two independent associations are 0.05 and 0.01, the joint significance is 0.0005^38^. Studies with multiple testing require statistical approaches to distinguish likely true associations from chance findings resulting from multiple tests, as has been described earlier^39^. In our study, we use different significance levels and joint p-values in order to differentiate the likely true associations from likely chance findings. We want to point out that the joint significance is used as a guidance for a Bonferroni-type adjustment as hundreds of comparisons were done. A single 95%CI is not very informative in the context of multiple comparisons. Statistical power was calculated to detect a significant association for two-sided 95% confidence level with an RR of 1.20 for one affected first-degree relative and an RR of 1.40 for at least two affected first-degree relatives. In the power calculation for bladder cancer and histology subtype analysis, the RR was set as 1.4. Trend tests were performed by modeling the number of first-degree relatives affected by cancer X as a continuous covariate. Potential confounders, including age group (17 groups with 5-year gap), sex, calendar period, residential area and socioeconomic status as well as parity for ovarian cancer risk, were added to the model as covariates. SAS version 9.4 was used to perform the statistical analysis.

### Ethical Statement

The study was approved by the Ethical Committee of Lund University without requirement for informed consent, and the study was conducted in accordance with the approved guidelines.

### Data Availability Statement

The data that support the findings of this study are available from Lund University but restrictions apply to the availability of these data, which were used under license for the current study and so are not publicly available.

## Electronic supplementary material

Supplementary Table S1 Familial associations of ovarian cancer with liver cancers

Supplementary Table S2 Familial associations of histology-specific ovarian cancers with gall bladder cancer

Supplementary Table S3 Risk of endometrioid ovarian cancer after diagnosis of endometrial cancer and risk of endometrial cancer after diagnosis of endometrioid ovarian cancer
