# Supplementary material for: Familial Ovarian Cancer Clusters with Other Cancers
Source: Sci Rep. 2018 Aug 1;8:11561. doi: 10.1038/s41598-018-29888-4 (PMC6070489; doi:10.1038/s41598-018-29888-4)
Supplement: Supplementary file 1 — Supplementary Table S1 Familial associations of ovarian cancer with liver cancers [file 41598_2018_29888_MOESM1_ESM.docx]

# FAMILIAL ASSOCIATIONS OF OVARIAN CANCER WITH OTHER CANCERS

Guoqiao Zheng ^1,2*^, Hongyao Yu^1,2^, Anna Kanerva ^3,4^, Asta Försti ^1,5^, Kristina Sundquist ^5,6,7^ and Kari Hemminki ^1,5^

^1^Division of Molecular Genetic Epidemiology, German Cancer Research Center (DKFZ), Heidelberg, Germany

^2^ Faculty of Medicine, University of Heidelberg, Heidelberg, Germany

^3^ Cancer Gene Therapy Group, Faculty of Medicine, University of Helsinki, Helsinki, Finland

^4^ Department of Obstetrics and Gynecology, Helsinki University Hospital, Helsinki, Finland

^5^ Center for Primary Health Care Research, Lund University, 205 02 Malmö, Sweden

^6^ Department of Family Medicine and Community Health, Department of Population Health Science and Policy, Icahn School of Medicine at Mount Sinai, New York, USA

^7^ Center for Community-based Healthcare Research and Education (CoHRE), Department of Functional Pathology, School of Medicine, Shimane University, Japan

*Corresponding author:

Guoqiao Zheng

Division of Molecular Genetic Epidemiology, German Cancer Research Center (DKFZ), Im Neuenheimer Feld 580, Heidelberg, D-69120, Germany

Phone: +49-6221-421805

Fax: +49-6221-421810

Email: [g.zheng@dkfz.de](mailto:g.zheng@dkfz.de)

Supplementary Table S1 Familial associations of ovarian cancer with liver cancers

| Subtypes | Risk of ovarian cancer | | | | Risk of liver cancers | | | |
| --- | --- | --- | --- | --- | --- | --- | --- | --- |
|  | *N* | *RR* | *95% CI* | *Power*  *(%)*  *RR=1.2* | *N* | *RR* | *95% CI* | *Power*  *(%)*  *RR=1.2* |
| Liver, primary | 92 | 1.05 | 0.85-1.29 | 26.3 | 74 | 1.07 | 0.85-1.35 | 11.0 |
| Gall bladder | 95 | **1.27** | 1.03-1.55 | 19.8 | 30 | 1.05 | 0.73-1.51 | 10.4 |
| Extrahepatic bile ducts | 33 | 1.37 | 0.97-1.93 | 11.2 | 23 | 1.47 | 0.97-2.22 | 7.1 |
| Ampulla of vater | 14 | 1.40 | 0.83-2.37 | 8.0 | 9 | 1.27 | 0.66-2.46 | 5.6 |

Bolding, italic and underlining indicate that the 95% CI, 99% CI and 99.9% CI did not overlap with 1.00 respectively;
