# Supplementary material for: Familial Ovarian Cancer Clusters with Other Cancers
Source: Sci Rep. 2018 Aug 1;8:11561. doi: 10.1038/s41598-018-29888-4 (PMC6070489; doi:10.1038/s41598-018-29888-4)
Supplement: Supplementary file 2 — Supplementary Table S2 Familial associations of histology-specific ovarian cancers with gall bladder cancer [file 41598_2018_29888_MOESM2_ESM.docx]

# FAMILIAL ASSOCIATIONS OF OVARIAN CANCER WITH OTHER CANCERS

Guoqiao Zheng ^1,2*^, Hongyao Yu^1,2^, Anna Kanerva ^3,4^, Asta Försti ^1,5^, Kristina Sundquist ^5,6,7^ and Kari Hemminki ^1,5^

^1^Division of Molecular Genetic Epidemiology, German Cancer Research Center (DKFZ), Heidelberg, Germany

^2^ Faculty of Medicine, University of Heidelberg, Heidelberg, Germany

^3^ Cancer Gene Therapy Group, Faculty of Medicine, University of Helsinki, Helsinki, Finland

^4^ Department of Obstetrics and Gynecology, Helsinki University Hospital, Helsinki, Finland

^5^ Center for Primary Health Care Research, Lund University, 205 02 Malmö, Sweden

^6^ Department of Family Medicine and Community Health, Department of Population Health Science and Policy, Icahn School of Medicine at Mount Sinai, New York, USA

^7^ Center for Community-based Healthcare Research and Education (CoHRE), Department of Functional Pathology, School of Medicine, Shimane University, Japan

*Corresponding author:

Guoqiao Zheng

Division of Molecular Genetic Epidemiology, German Cancer Research Center (DKFZ), Im Neuenheimer Feld 580, Heidelberg, D-69120, Germany

Phone: +49-6221-421805

Fax: +49-6221-421810

Email: g.zheng@dkfz.de

Supplementary Table S2 Familial associations of histology-specific ovarian cancers with gall bladder cancer

| Subtypes | Histology | Risk of ovarian cancer | | | | Risk of gall bladder cancer | | | |
| --- | --- | --- | --- | --- | --- | --- | --- | --- | --- |
|  |  | *N* | *RR* | *95% CI* | *Power*  *(%)*  *RR=1.4* | *N* | *RR* | *95% CI* | *Power*  *(%)*  *RR=1.4* |
| Gall bladder | Undifferentiated | 3 | 2.35 | 0.75-7.36 | 7.6 | 2 | ***6.95*** | 1.74-27.83 | 6.1 |
|  | Clear cell | 3 | 0.92 | 0.29-2.85 | 9.9 | 0 | - | - | 6.6 |
|  | Endometrioid | 7 | 1.09 | 0.52-2.29 | 12.7 | 2 | 1.62 | 0.40-6.49 | 7.8 |
|  | Serous | 35 | 1.24 | 0.89-1.73 | 27.3 | 9 | 1.78 | 0.93-3.44 | 12.3 |
|  | Mucinous | 12 | ***2.75*** | 1.56-4.88 | 11.1 | 4 | ***4.66*** | 1.75-12.42 | 7.4 |
|  | Non-epithelial | 3 | 1.94 | 0.62-6.07 | 8.5 | 0 | - | - | 6.3 |

Bolding, italic and underlining indicate that the 95% CI, 99% CI and 99.9% CI did not overlap with 1.00 respectively;
