# Supplementary material for: Familial Ovarian Cancer Clusters with Other Cancers
Source: Sci Rep. 2018 Aug 1;8:11561. doi: 10.1038/s41598-018-29888-4 (PMC6070489; doi:10.1038/s41598-018-29888-4)
Supplement: Supplementary file 3 — Supplementary Table S3 Risk of endometrioid ovarian cancer after diagnosis of endometrial cancer and risk of endometrial cancer after diagnosis of endometrioid ovarian cancer [file 41598_2018_29888_MOESM3_ESM.docx]

# FAMILIAL ASSOCIATIONS OF OVARIAN CANCER WITH OTHER CANCERS

Guoqiao Zheng ^1,2*^, Hongyao Yu^1,2^, Anna Kanerva ^3,4^, Asta Försti ^1,5^, Kristina Sundquist ^5,6,7^ and Kari Hemminki ^1,5^

^1^Division of Molecular Genetic Epidemiology, German Cancer Research Center (DKFZ), Heidelberg, Germany

^2^ Faculty of Medicine, University of Heidelberg, Heidelberg, Germany

^3^ Cancer Gene Therapy Group, Faculty of Medicine, University of Helsinki, Helsinki, Finland

^4^ Department of Obstetrics and Gynecology, Helsinki University Hospital, Helsinki, Finland

^5^ Center for Primary Health Care Research, Lund University, 205 02 Malmö, Sweden

^6^ Department of Family Medicine and Community Health, Department of Population Health Science and Policy, Icahn School of Medicine at Mount Sinai, New York, USA

^7^ Center for Community-based Healthcare Research and Education (CoHRE), Department of Functional Pathology, School of Medicine, Shimane University, Japan

*Corresponding author:

Guoqiao Zheng

Division of Molecular Genetic Epidemiology, German Cancer Research Center (DKFZ), Im Neuenheimer Feld 580, Heidelberg, D-69120, Germany

Phone: +49-6221-421805

Fax: +49-6221-421810

Email: g.zheng@dkfz.de

Supplementary Table S3 Risk of endometrioid ovarian cancer after diagnosis of endometrial cancer and risk of endometrial cancer after diagnosis of endometrioid ovarian cancer

| First primary | Second primary | N | Median (range) follow-up time /month | RR | 95% CI |
| --- | --- | --- | --- | --- | --- |
| Endometrioid ovarian cancer | Endometrial cancer | 40 | 0 (0, 5) | ***9.49*** | 6.96-12.95 |
| Endometrial cancer | Endometrioid ovarian cancer | 76 | 0 (0, 155) | ***20.30*** | 15.92-25.90 |

Bolding, italic and underlining indicate that the 95% CI, 99% CI and 99.9% CI did not overlap with 1.00 respectively;
